# Underhydration Is Associated with Obesity, Chronic Diseases, and Death Within 3 to 6 Years in the U.S. Population Aged 51–70 Years

**DOI:** 10.3390/nu12040905

**Published:** 2020-03-26

**Authors:** Jodi D. Stookey, Stavros A. Kavouras, HyunGyu Suh, Florian Lang

**Affiliations:** 1 Hydration Science Lab, College of Health Solutions, Arizona State University, Phoenix, AZ 85004, USA; Stavros.kavouras@asu.edu (S.A.K.); HyunGyu.Suh@asu.edu (H.S.); 2 Department of Physiology, Eberhard Karls University, Tubingen 72074, Germany; florian.lang@uni-tuebingen.de

**Keywords:** hydration, chronic disease, mortality

## Abstract

Nationally representative data from the National Health and Nutrition Examination Survey (NHANES) indicate that over 65% of adults aged 51–70 years in the U.S. do not meet hydration criteria. They have hyponatremia (serum sodium < 135 mmol/L) and/or underhydration (serum sodium >145 mmol/L, spot urine volume <50 mL, and/or spot urine osmolality ≥500 mmol/kg). To explore potential public health implications of not meeting hydration criteria, data from the NHANES 2009–2012 and National Center for Health Statistics Linked Mortality Files for fasting adults aged 51–70 years (sample *n* = 1200) were used to determine if hyponatremia and/or underhydration were cross-sectionally associated with chronic health conditions and/or longitudinally associated with chronic disease mortality. Underhydration accounted for 97% of the population group not meeting hydration criteria. In weighted multivariable adjusted Poisson models, underhydration was significantly associated with increased prevalence of obesity, high waist circumference, insulin resistance, diabetes, low HDL, hypertension, and metabolic syndrome. Over 3–6 years of follow-up, 33 chronic disease deaths occurred in the sample, representing an estimated 1,084,144 deaths in the U.S. Alongside chronic health conditions, underhydration was a risk factor for an estimated 863,305 deaths. Independent of the chronic health conditions evaluated, underhydration was a risk factor for 128,107 deaths. In weighted multivariable Cox models, underhydration was associated with 4.21 times greater chronic disease mortality (95% CI: 1.29–13.78, *p* = 0.019). Zero chronic disease deaths were observed for people who met the hydration criteria and did not already have a chronic condition in 2009–2012. Further work should consider effects of underhydration on population health.

## 1. Introduction

Data from the 2009–2012 National Health and Nutrition Examination Survey (NHANES) indicate that the majority of adults ages 51–70 years in the U.S. do not meet hydration criteria [1]. An estimated 72.1% (68.4%–75.5%) of men and 65.9% (61.0%–70.6%) of women who are free of acute illness have a serum sodium below 135 mmol/L, a serum sodium of 145 mmol/L or higher, a spot urine volume less than 50 mL, or a urine osmolality of 500 mmol/kg or higher. Individuals who do not meet these hydration criteria have hyponatremia and/or a condition described as “underhydration” by Kavouras [2], which is characterized by elevated vasopressin and renal conservation of water despite normal plasma osmolality. The 2009–2012 NHANES analysis [1] has implications for national water intake recommendations, if hyponatremia and/or underhydration signal risk for poor health outcomes. Given controversy over hydration criteria [3] and no population-representative estimates of health benefit associated with euhydration as defined by Stookey [1], this analysis extends work on the same 2009–2012 NHANES study population to describe the prevalence of chronic health conditions and risk of mortality associated with not meeting the same hydration criteria.

### 1.1. Hydration Classification

The public health significance of not meeting the hydration criteria specified by Stookey [1] is uncertain, because it is possible to define hydration relative to various standards and index hydration using multiple different measures, either alone or in combination. Hydration may be defined relative to requirements for various aspects of metabolism, such as sodium balance, protein turnover, and hormone release and action, requirements for various aspects of physiological homeostasis, such as maintenance of blood volume, blood pressure, and thermoregulation; requirements for various aspects of physical function or performance, such as urine concentration, endurance, and cognition; and/or myriad other requirements for health. Hydration measures, such as serum sodium, blood urea nitrogen creatinine ratio, anti-diuretic hormone, hematocrit, heart rate, urine concentration, sweat rate, skin turgor, and others, have been developed to reflect one or more of the above aspects of metabolism, physiology, function, or health [4,5,6]. No one gold standard of hydration has been identified [7,8]. Available hydration indices vary in sensitivity and specificity to a range of mild to severe, acute to chronic, fluid disorders, including hyper-, iso-, and hypovolemic dehydration. The hydration indices vary in terms of prevalence under conditions of daily life [9]. Assessment of one or more hydration measures may be appropriate to address any given public health question.

### 1.2. Cross-Sectional Association between Hydration Classification and Chronic Health Conditions

The 2009–2012 NHANES data suggest that the high prevalence of not meeting the hydration criteria is associated with risk of chronic disease. Compared with the prevalence estimated for the total population of non-acutely ill men and women ages 51–70 years (noted above), the prevalence estimated for the sub-group without chronic disease risk factors is over 15 percentage points lower (52.4% (40.1%–64.4%) for males, 49.0% (34.3%–63.9%) for females) [1]. The chronic disease risk factors considered in the analysis are underweight, overweight or obesity, hyperglycemia, insulin resistance, hypertriglyceridemia, low high-density lipoprotein (HDL), and chronic kidney disease.

Individuals with chronic disease are more likely to have hyponatremia than individuals without chronic disease. NHANES data from 1999–2004 indicate that, in U.S. adults, hyponatremia is significantly more common among those with hypertension, diabetes, coronary artery disease, stroke, chronic obstructive pulmonary disease, cancer, and psychiatric disorders, and less common in those with no comorbidities. Controlling for demographics, smoking, comorbidities, and insurance, hyponatremia more than doubles the risk of death within 3 to 6 years [10].

Individuals with chronic disease are also at increased risk of underhydration. At a given point in time, conditions of the metabolic syndrome predispose to higher serum sodium, low urine volume, and/or concentrated urine. In cross-sectional data, obese individuals are significantly more likely to have a serum sodium above 140 mmol/L and an increased extracellular relative to intracellular (ECF/ICF) fluid ratio than normal weight individuals [11]. Insulin resistance causes extracellular glucose to become osmotically effective, favoring osmotic cell shrinkage, vasopressin release, and vasopressin action on the kidney to reduce urine volume and concentrate urine [12,13,14]. Impaired insulin-stimulated glucose metabolism and salt-sensitive hypertension occur with renal sodium retention [15,16,17]. Plasma copeptin, a biomarker of osmotic shrinkage of cells and vasopressin release [18], is cross-sectionally associated with diabetes and insulin resistance [19]. Higher systolic blood pressure is cross-sectionally associated with higher serum sodium [20,21,22,23,24]. Across studies, individuals with metabolic syndrome have significantly higher levels of sodium compared to healthy controls [25].

Cross sectional associations are not informative about the temporality of association. Although chronic health conditions may increase risk of underhydration, the reverse is also possible. Underhydration status may determine chronic health conditions. Prospectively, hypernatremia, low urine volume, and/or concentrated urine predict the risk of new onset or worsening metabolic dysregulation. In controlled experiments, increasing the serum sodium over 145 mmol/L of healthy adults via fluid restriction or hypertonic saline infusion decreases insulin sensitivity, increases hyperglycemia, and alters lipid metabolism [26,27,28]. At the cell level, experiments show that extracellular hypertonicity causes cell shrinkage, which upregulates proteolysis and glycogenolysis [29]. The free amino acids and glucose end-products, in turn, trigger insulin release. Extracellular hypertonicity, furthermore, causes insulin resistance by counteracting cell swelling, which is required for the effect of insulin on metabolism, such as proteolysis [30]. Sustained over time, extracellular hypertonicity causes cells to alter gene transcription to support cellular accumulation of organic osmolytes [31]. Over the longer term, higher serum sodium predicts many age-related degenerative diseases [32].

### 1.3. Longitudinal Association between Hydration Classification and Mortality

Longitudinal, population-representative data are consistent with a negative feedback system of not meeting hydration criteria and metabolic dysregulation that magnifies risk of incident disease. As the number of metabolic syndrome components increases, the body sodium level increases [25]. In people with hyperglycemia, independent of the level of plasma glucose, a unit increase in serum sodium increases the relative odds of incident diabetes by 26% [33]. In people with diabetes, independent of fasting glucose and hemoglobin A1c, plasma copeptin significantly interacts with diabetes to predict 33% greater risk of coronary artery disease, 62% greater risk of heart failure, and 32% greater risk of death from diabetes [34]. Longitudinal data indicate significantly greater mortality when serum sodium is outside the normal range or plasma copeptin or urine osmolality is elevated [10,19,35,36,37,38,39,40,41,42,43,44,45].

The ultimate goal of this analysis was to facilitate interpretation of recent results from the 2009–2012 NHANES [1]. To align with that analysis, the specific aims of this analysis were to determine if “not meeting hydration criteria”, as defined in that analysis [1], was (1) cross-sectionally associated with chronic health conditions in 2009–2012 and (2) longitudinally associated with death within 3–6 years. The cross-sectional analysis checked for potential intercorrelation or effect mediation between hydration classification and chronic health conditions in 2009–2012. The survival analysis specified the joint effect of baseline hydration and chronic health condition as main exposure.

Given the working hypothesis that hyponatremia and/or underhydration exacerbate metabolic dysregulation characteristics of metabolic syndrome, this analysis also reports cross-sectional associations between underhydration and chronic health conditions. Cross-sectional associations between hyponatremia and chronic health conditions have already been described for the NHANES population [10].

## 2. Materials and Methods

### 2.1. Study Design

Data from the 2009–2010 and 2011–2012 National Health and Nutrition Examination Survey (NHANES) were pooled for cross-sectional analysis of prevalent chronic health conditions in 2009–2012 and prospective analysis of mortality risk from 2009–2012 through December 31, 2015. NHANES collects information about demographic, behavioral, and biological risk factors via home interview, physical examination, dietary assessment, and laboratory tests. For the prospective analysis, NHANES data were linked to death records from the National Death Index using National Center for Health Statistics Linked Mortality Files [46].

### 2.2. Study Population

NHANES selects a stratified, multistage probability sample which is representative of the civilian non-institutionalized U.S. population [47]. The present study population was selected to match a study population previously described [1], which included non-acutely ill men and women aged 51–70 years who had valid information about water intake, body weight, and serum sodium. The present study population excluded individuals with extreme serum creatinine (< 0.5 or >1.5 mg/dL; *n* = 75) or albuminuria (urine albumin/creatinine ratio ≥30 mg/g creatinine; *n* = 424), for whom hydration status might be misclassified. The study population excluded 1335 individuals who were not 8 h fasted at the time of the 2009–2012 examination, as fasting laboratory test results were used to classify study participants with respect to chronic conditions. Participants missing information about chronic disease conditions (*n* = 41) and vital status through 2015 (*n* = 2) were excluded. The analysis sample included 1200 individuals aged 51–70 years.

The NHANES 2009–2010 and 2011–2012 protocols were reviewed and approved by the Centers for Disease Control and Prevention (CDC) National Center for Health Statistics (NCHS) Institutional Review Board (IRB) / Ethics Review Board (ERB) (https://www.cdc.gov/nchs/nhanes/irba98.htm). All study participants provided informed consent. The study complied with the Declaration of Helsinki for human subject involved medical research.

### 2.3. Measures

#### 2.3.1. Hydration

A fasting blood and spot urine sample were collected and processed at the mobile examination center (MEC) following protocol described in detail elsewhere [1,48,49]. Serum sodium was determined as part of a DxC800 serum biochemistry profile [50,51]. Urine osmolality was determined by freezing point depression osmometer (Osmette II, Model 5005 Automatic Osmometer, Precision Systems Inc, Natick, MA, USA) [52].

Fasting serum sodium, spot urine volume, and spot urine osmolality were used to classify study participants according to previously defined hydration criteria [1,53,54], which are consistent with zero electrolyte free water clearance [55], proposed cutoffs for urine osmolality [56,57], and possible chronic health benefits [58,59,60]. For urine osmolality, the cutoff of 500 mmol/kg was selected because of its sensitivity to cell swelling, suppressed vasopressin release, and urine dilution. Due to the tight relationship between cell shrinkage and vasopressin release [61], higher urine osmolality cutoffs distinguish cell shrinkage and urine concentration.

Participants who met all criteria were identified as euhydrated. Participants who did not meet any one of the criteria were classified as not meeting the hydration criteria. Participants who were unable to void, a sign of vasopressin activation and action, were classified with participants with urine volume below 50 mL, as not meeting the hydration criteria. Participants that did not meet criteria and did not have hyponatremia were classified as underhydrated.

#### 2.3.2. Chronic Conditions

Measured body weight, height, and waist circumference as well as CDC cutoffs (body mass index (BMI) ≥ 30; waist > 40 inches for men, waist > 35 inches for women) were used to classify the weight status of participants [62].

Consistent with previous NHANES analyses [1,63], fasting insulin determined by enzyme-linked immunosorbent assay (ELISA), fasting glucose determined by enzymatic method, and the Homeostatic Model Assessment for Insulin Resistance (HOMA-IR) equation (with ≥2.5 cutoff) were used to classify participants with respect to insulin resistance. Fasting glucose and glycosylated hemoglobin (HbA1c) were classified as elevated following American Diabetes Association cutoffs (glucose ≥ 126 mg/dL; HbA1c ≥ 6.5%) [64]. HbA1c was determined by a laboratory affiliated with the National Glycohemoglobin Standardization Program using a Tosoh A1C G7 system [64,65]. Participants who had an elevated fasting glucose, elevated HbA1c or an oral glucose tolerance test result at or above 200 mg/dL, or who reported ever being told that they had diabetes were classified as having diabetes.

Consistent with the previous NHANES analysis [1], fasting triglycerides above 150 mg/dL were considered elevated [53,66,67]. Fasting HDL cholesterol below 40 mg/dl for men and below 50 mg/dL for women were considered low [53,66,67].

Participants were classified as having hypertension on the basis of blood pressure measurements taken at the MEC visit and American Heart Association cutoffs (systolic blood pressure (SBP) ≥ 120, diastolic blood pressure (DBP) ≥ 80) [68] or ever diagnosis of high blood pressure [69].

National Cholesterol Education Program, Adult Treatment Panel III (NCEP/ATPIII) criteria were used to classify participants with respect to metabolic syndrome (three or more of the following conditions: high waist circumference, elevated triglycerides, blood pressure ≥130/ ≥ 85 mmHg, low HDL (< 40 mg/dL for men and <50 mg/dL for women), and fasting glucose (≥ 110 mg/dL) [70]. Participants with none of the above conditions were identified (BMI, waist circumference, HOMA-IR, fasting glucose, HbA1c and fasting triglyceride below cutoffs, no diabetes, no hypertension and no metabolic syndrome).

#### 2.3.3. Joint Exposure

Given potential for a feedback system between hydration and metabolic dysregulation, participants were divided into six groups defined by euhydration, hyponatremia, or underhydration and whether or not they had any of the chronic health conditions (yes/no) at baseline. Participants with underhydration AND any of the chronic conditions at baseline were considered further progressed along the feedback system of metabolic dysregulation than participants with underhydration OR any of the chronic conditions at baseline. The latter groups, in turn, were considered further progressed along the feedback system than participants who were euhydrated and did not have any of the chronic conditions in 2009–2012.

#### 2.3.4. Mortality

Survival time was expressed as the number of months elapsed between the MEC examination in 2009–2012 and December 31, 2015. Vital status was expressed as deceased or assumed alive through December 31, 2015. The underlying cause of death was classified as heart disease (I00-I09, I11, I13, I20-I51), malignant neoplasm (C00-C97), or all other causes, on the basis of the 10th International Statistical Classification of Diseases and Related Health Problems (ICD-10) codes. Deaths with hypertension and/or diabetes listed as a contributing cause were flagged. Deaths with heart disease, malignant neoplasms, hypertension, and/or diabetes listed as an underlying cause or multiple cause were grouped.

#### 2.3.5. Covariates

Age in years, sex, and height measured at the MEC (as proxy for body size) were considered as determinants of individual water intake requirements [71]. Socio-economic covariates included self-reported race/ethnicity (non-Hispanic black, Hispanic, white and other/multi-racial), education (less than high school diploma, high school or General Educational Development (GED), college or postgraduate), and poverty (percent of Federal Poverty Level (FPL)). Season of the MEC examination (winter or spring, summer or fall) was considered as proxy for ambient temperature and environmental determinant of both water intake and water intake requirements. The total length of food fast was measured as the number of hours between the last food or drink consumed, other than water, and the venipuncture.

Behavioral covariates included self-reported prescription medication use in the past month [72,73], lifetime smoking experience [74,75], physical activity in the past 7 days, and dietary intake in the 24 h before the morning blood and urine sample collection.

To index physical activity in the past 7 days, a trained interviewer administered a Global Physical Activity Questionnaire (GPAQ) using the computer-assisted personal interviewing (CAPI) system [76,77]. Participants were asked to self-report how much time they spent walking, biking, or doing moderate or vigorous activities at work and/or for recreation on each day in the previous week. The time estimates were converted to metabolic equivalents (MET) minutes per week, assuming a MET score of 4.0 for moderate physical activity and MET score of 8.0 for vigorous activities.

The previous day total intakes of energy, carbohydrate, alcohol, and solute load were estimated from an in-person 24 h dietary recall, administered by a trained interviewer using the United States Department of Agriculture (USDA) Automated Multiple Pass software and portion size reference guides [78,79]. Interviewers probed for snacks and drinks between meals and food preparation details, including addition of salt and sugar during cooking or at the table. Total carbohydrate intake was expressed as a continuous variable relative to total energy intake. Alcohol was expressed in categorical terms, as no drinks, 1–2 drinks, or 3 or more drinks per day. Total daily intakes of protein, sodium, and potassium were combined to index dietary solute load: (gm protein x 5.8) + mEq (Na + K). The calculated index underestimates the total solute load, because solute, such as chloride, were not available in the dataset.

### 2.4. Statistical Analysis

Statistical analyses were performed using Stata/SE 15.1 statistical software (StataCorp LP; College Station, TX, USA), pooled weights for the 2009–2010 and 2011–2012 NHANES fasting sub-samples (1/2*wtsaf2yr) [1,80], and a *p*-value <0.05 for statistical significance.

#### 2.4.1. Descriptive Analysis

The bivariate relationship between serum sodium and urine osmolality in the study sample was described by scatterplot. The estimated population-representative distributions of serum sodium, urine osmolality, and urine volume in the study population were described using weighted geometric means and 95% confidence intervals. The estimated population-representative prevalence of euhydration and not meeting the hydration criteria in the study population were described using weighted proportion and 95% confidence intervals.

The study population was described in terms of risk factors that might confound effects of hydration on chronic disease risk, namely, socio-economic, environmental, and behavioral determinants of water intake requirements and water intake. The weighted proportion of the study population with each risk factor was estimated by hydration classification. Unadjusted weighted Poisson models with robust error variances tested for association between each risk factor and hydration classification.

#### 2.4.2. Cross-Sectional Relationship between Chronic Disease and Hydration Classification

Assuming that chronic health conditions may increase risk of not meeting the hydration classification and, conversely, that not meeting the hydration classification may increase risk of having a chronic condition, effects in both directions were estimated.

The weighted prevalence of not meeting the hydration criteria was estimated, stratified by chronic health condition. Unadjusted and multi-variable adjusted weighted Poisson models with robust error variances were used to test if the prevalence ratio of not meeting the hydration criteria in 2009–2012 varied by health condition in 2009–2012. A first multivariable model (Model 2) controlled for age, sex, height, race–ethnicity, education, poverty level, physical activity, total energy intake, total carbohydrate intake, alcoholic drinks consumed, dietary solute load, past or current cigarette smoking, prescription medication use, season of MEC measurement, and length of fast prior to venipuncture. A second multivariable model (Model 3) added control for the other chronic conditions (obesity, insulin resistance, diabetes, dyslipidemia, and hypertension) to check if metabolic dysregulation related to concurrent chronic conditions might mediate or explain the association. To explore for effects specific to underhydration, a set of models with the same specification were fit to test if the prevalence ratio of underhydration varied by chronic health condition.

The weighted prevalence of each chronic condition was next estimated, stratified by hydration classification. Unadjusted and multi-variable adjusted weighted Poisson models with robust error variances were used to test if the prevalence ratio of having each chronic condition in 2009–2012 varied by hydration classification in 2009–2012 (not meeting the hydration criteria vs. euhydration as well as underhydration vs. euhydration). Multivariable models adjusted for covariates, as described above.

#### 2.4.3. Longitudinal Effect of Hydration Classification on Death

The weighted number of deaths occurring to the study population within 3 to 6 years of the MEC examination was estimated, stratified by hydration classification in 2009–2012, health condition in 2009–2012, and all-cause or chronic disease-associated death. Health condition was defined as any or none of the following health conditions: obesity, high waist circumference, insulin resistance, diabetes, elevated glucose, HgA1c or triglycerides, low HDL, hypertension, or metabolic syndrome. All-cause and chronic disease-associated death rates were estimated with jackknife confidence intervals. The stset command available in stata was used for survival analyses with a time-on-study scale [81] and failure defined as ‘all-cause death’ or ‘chronic-disease associated death’. Kaplan–Meier survival curves were graphed by hydration classification as well as by the joint exposure of hydration classification and chronic health condition at baseline.

**Figure 1:**
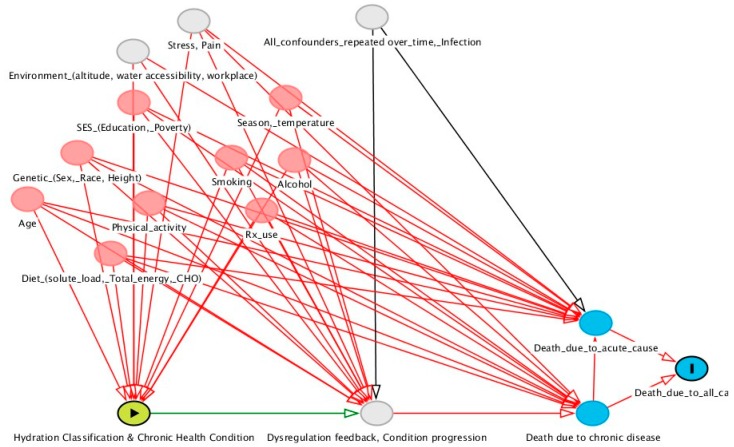
Directed acylic graph (DAG) representing the hypothesized joint effect of hydration classification and chronic health condition on death. The yellow circle highlights the joint exposure. The blue circles represent the outcome. The pink and grey circles represent covariates that were observed and unobserved by National Health and Nutrition Examination Survey (NHANES), respectively. The DAG assumes that the hydration classification is a function of water intake requirements, water intake, and health condition. Many unobserved covariates and interrelationships between covariates are not shown. Figure 1 was generated using DAGgity.net v2.3.

Weighted Cox proportional hazards models were used to test for differences in the hazard rate by hydration classification. All multivariable models controlled for all covariates described above for Model 2. The aim of this descriptive analysis was to determine if euhydration, as defined previously [1], signals significantly lower mortality or not. The aim was not to estimate an independent causal effect of hydration, as some confounding and time-varying variables were not available in NHANES (see Figure 1). A first set of unadjusted and multivariable-adjusted models estimated the mortality risk associated with euhydration vs. not meeting hydration criteria [1]. A second set of models estimated the mortality risk associated with euhydration vs. underhydration, more specifically.

If hydration and chronic health condition(s) interact in a negative feedback system, then effects of health condition(s) on mortality would be expected to be worse if hydration criteria are not met. To check for this possibility, unadjusted and multivariable adjusted weighted Cox proportional hazards models tested for greater risk of mortality associated with not meeting the hydration criteria among individuals with one or more chronic health conditions in 2009–2012.

#### 2.4.4. Sensitivity Analyses

The hydration classification exposure selected for this analysis was defined to account for U-shaped hydration effects and distinguish euhydration from hyponatremia as well as underhydration. To illustrate need to account for U-shaped hydration effects, Poisson models, specified as described above, tested for a significant U-shaped relationship between serum osmolality and mortality. Serum osmolality was calculated from serum chemistry results as 1.86 × (Na+ + K+) + 1.15 × glucose + urea + 14 [82]. The models included a main effect for serum osmolality and serum osmolality*serum osmolality interaction term. The sample for this sensitivity analysis included adults aged 71–80 years.

The specific aims of this paper were focused on the 51–70-year-old age group to align with the previous analysis [1] as well as account for potential effect modification by older age related to factors such as decreased urine concentrating ability and/or increased risk of isotonic hypovolemia with age [1]. Unadjusted and multivariable-adjusted weighted models tested whether age group (71–80 vs. 51–70 years) modified the effect of underhydration on chronic disease mortality. The sample for this sensitivity analysis included adults aged 71–80 years.

Beyond accounting for U-shape, the hydration classification exposure selected for this analysis was assumed to reflect biologically meaningful variability that serum osmolality masks. Serum osmolality may be normal at the expense of vasopressin-mediated conservation of body water. Vasopressin-mediated pathways are associated with increased chronic disease mortality [19,34,37]. To check this working assumption, additional multivariable adjusted weighted Poisson models tested for an effect of underhydration on chronic disease mortality for persons aged 51–70 years with a calculated serum osmolality in the normal range, between 285 and 295 mmol/kg [82].

Finally, to consider if underhydration effects on chronic disease mortality might conceivably be attributable to vasopressin-mediated conservation of body water, as opposed to underlying diabetes by itself (elevated urine osmolality due to glycosuria), a last set of unadjusted and multivariable weighted adjusted Poisson models tested for a difference in the hazard between low urine volume and euhydration for individuals with urine osmolality below 500 mmol/kg.

## 3. Results

### 3.1. Sample Characteristics

**Figure 2:**
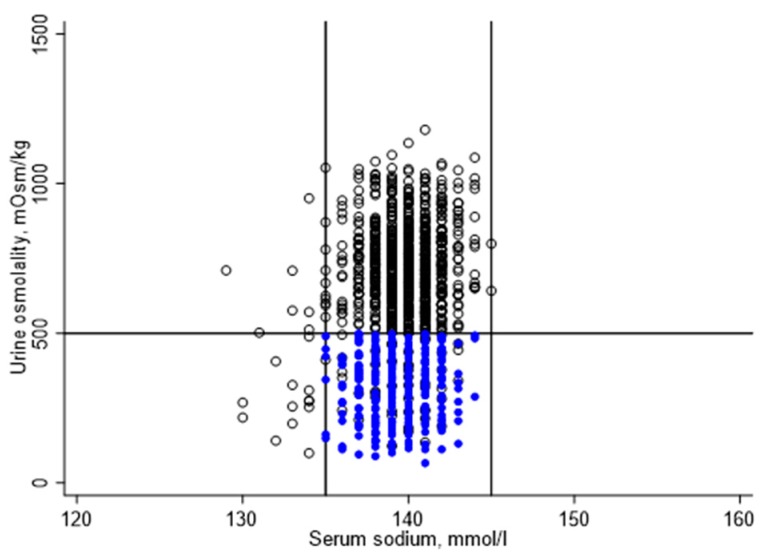
Distribution of serum sodium and urine osmolality in the study sample.

Figure 2 illustrates the distribution of serum sodium and urine osmolality for the study sample. Serum sodium ranged from 129 to 145 mmol/L, with a mean (standard error (SE)) of 139 (0.1) mmol/l. Urine osmolality ranged from 66 to 1180 mmol/kg, with a mean (SE) of 537 (11) mmol/kg. Urine volume ranged from 1 to 466 mL, with a mean (SE) of 77 (3) mL.

Of 1200 study participants, 881 did not meet one or more hydration criteria. A total of 22 participants were hyponatremic, 10 of which had a urine osmolality below 500 mmol/kg. A total of 859 participants were classified as underhydrated, including 322 participants with zero urine or a urine volume below 50 mL. Urine osmolality was 500 mmol/kg or higher for 796 participants, including 537 participants with urine volume of 50 mL or more.

Applying sample weights, an estimated 71.4% (67.0%–75.4%) of fasting U.S. adults aged 51–70 years in 2009–2012 did not meet one or more hydration criteria. Among those who did not meet the hydration criteria, 2.7% (1.3%–5.5%) had hyponatremia and 97.3% (94.5%–98.7%) were underhydrated. An estimated 69.4% (65.5%–73.1%) of the study population was underhydrated.

Table 1 describes the hydration classification by risk factors for chronic health conditions and/or mortality. Compared to euhydration, the prevalence of not meeting the hydration criteria varied significantly by race/ethnicity, cigarette smoking, total daily dietary solute load, total energy, and carbohydrate intake.

### 3.2. Prevalence of Not Meeting Hydration Criteria by Chronic Health Condition

Table 2A describes the number of NHANES participants and weighted proportion of the study population who did not meet one or more hydration criteria, stratified by chronic condition. The table aligns with groups defined in the prior NHANES analysis [1]. Independent of the covariates controlled in Model 2, the prevalence of not meeting hydration criteria was significantly higher among individuals with obesity, high waist circumference, insulin resistance, low HDL, and metabolic syndrome than among individuals without these conditions. Except for the relationship between obesity and not meeting hydration criteria, the observed associations were explained away by control for other chronic conditions (Model 3).

Whereas Table 2A describes the prevalence of not meeting one or more hydration criteria, Table 2B focuses on the prevalence of underhydration, more specifically, excluding hyponatremia. The same pattern of results was observed for underhydration. Independent of the covariates controlled in Model 2, the prevalence of underhydration was significantly higher among individuals with obesity, high waist circumference, insulin resistance, low HDL, and metabolic syndrome than among individuals without these conditions.

### 3.3. Prevalence of Chronic Health Condition by Hydration Classification

Table 3A,B describes the number of NHANES participants and weighted prevalence of each chronic health condition, stratified by hydration classification. As above, Table 3A describes associations for not meeting one or more of the hydration criteria, whereas Table 3B focuses on underhydration specifically. In unadjusted weighted Poisson models, obesity, high waist circumference, insulin resistance, diabetes, low HDL, hypertension, and metabolic syndrome were significantly more prevalent among individuals who did not meet hydration criteria. The proportion of people without any chronic condition was two times greater for people who were euhydrated. The relationships remained statistically significant after adjustment for all covariates in Model 2. The multivariable-adjusted prevalence ratio for each condition (Model 2) was reduced by more than 10% after further adjustment for all other chronic conditions (Model 3).

### 3.4. Mortality Associated with Hyponatremia or Underhydration vs. Euhydration

#### 3.4.1. All-Cause Mortality

**Figure 3:**
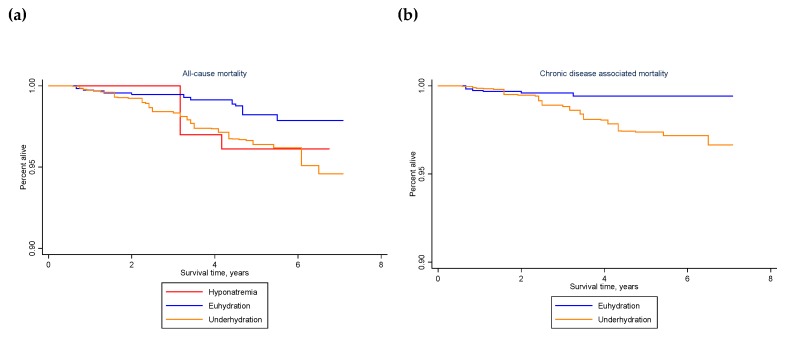
Kaplan–Meier curves for (**a**) all-cause and (**b**) chronic disease-associated mortality associated with hyponatremia, euhydration, or underhydration in 2009–2012 for U.S. adults aged 51–70 years. Hyponatremia: serum sodium below 135 mmol/L; euhydration: serum sodium 135–144 mmol/L, urine volume 50 mL or higher, and urine osmolality below 500 mmol/kg; underhydration: serum sodium 145 mmol/L or higher, urine volume below 50 mL, or urine osmolality 500 mmol/kg or higher.

Table 4 summarizes the number of deaths that occurred within 3 to 6 years of the 2009–2012 MEC examination. A total of 52 deaths in the 2009–2012 NHANES study sample represented 1,724,051 deaths to persons ages 51–70 years, nationally. The observed deaths were concentrated among persons who were underhydrated in 2009–2012. Figure 3 (a) illustrates the difference in survival for people who were hyponatremic or underhydrated vs. euhydrated. The unadjusted all-cause death rate was over two times higher for people who did not meet the hydration criteria in 2009–2012 (weighted hazard ratio (HR): 2.37, 95% confidence interval (CI): 1.02–5.50, *p* = 0.044), either because of hyponatremia (weighted HR: 2.32, 95% CI: 0.32–16.89, *p* = 0.396) or underhydration (weighted HR: 2.37, 95% CI: 1.03–5.49, *p* = 0.044). The estimated increase in all-cause mortality associated with not meeting hydration criteria remained over two times higher after adjustment for covariates (adjusted weighted HR: 2.32, 95% CI: 0.96–5.63, *p* = 0.062). The estimated increase in all-cause mortality associated with underhydration, more specifically, also remained over two times higher (adjusted weighted HR: 2.33, 95% CI: 0.97–5.60, *p* = 0.059).

#### 3.4.2. Chronic Disease Mortality

An estimated 1,084,144 deaths in the study population were attributed to or associated with heart disease, malignant neoplasms, hypertension, and/or diabetes. The unadjusted risk of chronic disease-associated death was about four times higher for underhydrated vs. euhydrated people in 2009–2012 (unadjusted weighted HR: 4.44, 95% CI: 1.57–12.54, *p* = 0.006). After adjustment for covariates, underhydration remained significantly associated with chronic disease-associated death (adjusted weighted HR: 4.21, 95% CI: 1.29–13.78, *p* = 0.019). Figure 3 (b) illustrates the difference in survival.

#### 3.4.3. Sensitivity Analyses

For adults aged 51–80 years, a U-shaped relationship was observed between serum osmolality and all-cause as well as chronic disease mortality. For the 51–70-year-old age group, a U-shaped relationship was observed between serum osmolality and all-cause, but not chronic disease mortality (see Table A1). The tails of the serum osmolality distribution, below 285 mmol/kg and above 300 mmol/kg, were associated with the increased risk of chronic disease mortality (see Table A2).

The effect of underhydration on chronic disease associated mortality was significantly modified by age group (adjusted weighted HR (SE) for the interaction term: 0.23 (0.17), *p*-value = 0.048).

For adults aged 51–70 years with a serum osmolality between 285–295 mmol/kg, underhydration was associated with significantly increased risk of chronic disease mortality (unadjusted weighted HR: 3.52, 95% CI: 1.12–11.10, *p* = 0.032; adjusted weighted HR: 3.45, 95% CI: 0.94–12.64, *p* = 0.061).

Focusing on underhydrated individuals with urine osmolality below 500 mmol/kg, the unadjusted and multivariable-adjusted relative hazards for chronic disease mortality associated with low urine volume vs. euhydration were as follows: unadjusted weighted HR: 7.86, 95% CI: 1.14–54.38, *p* = 0.037; adjusted weighted HR: 13.32, 95% CI: 1.01–176.25, *p* = 0.049.

### 3.5. Mortality Associated with the Joint Effect of Hydration Classification and Chronic Health Condition

Across the different chronic health conditions in 2009–2012, deaths were concentrated among those who were underhydrated. Health condition-specific data were not reported because of small numbers. There were fewer than 10 chronic disease-associated deaths in the euhydrated group.

**Figure 4:**
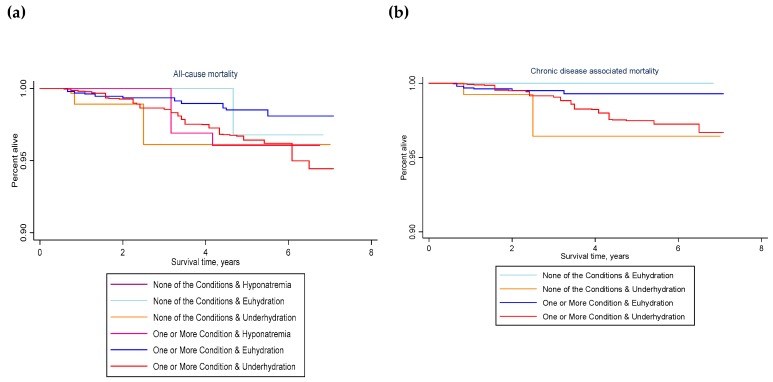
Kaplan–Meier curves for (**a**) all cause and (**b**) chronic disease-associated mortality by chronic health condition and hydration classification in 2009–2012 for U.S. adults aged 51–70 years. Hyponatremia: serum sodium below 135 mmol/L; euhydration: serum sodium 135–144 mmol/L, urine volume 50 mL or greater, and urine osmolality below 500 mmol/kg; underhydration: serum sodium 145 mmol/L or higher, urine volume below 50 mL, or urine osmolality 500 mmol/kg or higher. Chronic health conditions: obesity, high waist circumference, insulin resistance, diabetes, elevated glucose, elevated HgA1c, elevated triglycerides, low HDL, hypertension, and metabolic syndrome in 2009–2012.

Table 4 summarizes the number of deaths that occurred by presence or absence of chronic health condition in 2009–2012. Figure 4 describes survival by the joint exposure of hydration classification and chronic health condition in 2009–2012.

Among those with one or more chronic health conditions in 2009–2012, compared to euhydration, not meeting hydration criteria was associated with significantly greater risk of all-cause mortality within 3 to 6 years (unadjusted weighted HR: 2.51, 95% CI: 1.14–5.51, *p* = 0.024; adjusted weighted HR: 2.50, 95% CI: 1.07–5.85, *p* = 0.036). Compared to euhydration, underhydration was associated with significantly greater all-cause mortality (unadjusted weighted HR: 2.51, 95% CI: 1.13–5.54, *p* = 0.025; adjusted weighted HR: 2.47, 95% CI: 1.06–5.71, *p* = 0.036).

Among those with one or more chronic health conditions in 2009–2012, the risk of death attributed to chronic disease was over three times greater for people who were underhydrated vs. euhydrated (unadjusted weighted HR: 3.51, 95% CI: 1.17–10.53, *p* = 0.027; adjusted weighted HR: 3.08, 95% CI: 0.92–10.32, *p* = 0.067).

It was not possible to estimate the effect of underhydration on mortality for participants with none of the chronic conditions at baseline, due to the small number of deaths in this group. No chronic disease-associated deaths were observed during the follow-up period for people who did not have any of the chronic conditions and were, also, euhydrated in 2009–2012. Compared with people who did have one or more chronic condition, but were classified as euhydrated, in 2009–2012, people who were not identified as having any chronic condition at baseline but were underhydrated were significantly more likely to die (unadjusted weighted HR: 5.41, 95% CI: 1.32–22.15, *p* = 0.020; adjusted weighted HR: 8.43, 95% CI: 1.15–61.75, *p* = 0.037).

## 4. Discussion

The results of this study suggest that over 95% of U.S. adults aged 51–70 years who do not meet hydration criteria are underhydrated. The results further suggest that underhydration signals a statistically significant health risk. Underhydration, that is, serum sodium above the 135–144 mmol/L range, spot urine volume less than 50 mL, or urine osmolality of 500 mmol/kg or higher, was cross-sectionally associated with increased prevalence of multiple chronic health conditions. Underhydration was longitudinally associated with increased risk of all-cause and chronic disease associated mortality within 3–6 years.

### 4.1. Hydration Classification

Researchers debate which biomarker to use for epidemiologic analysis, dietary recommendations, and public health policy. Various hydration biomarkers have been developed to index a variety of body fluid disorders [7,83,84]. Any one biomarker may not be optimally sensitive to all types of body fluid disorders [9]. This analysis explored the public health significance of not meeting hydration criteria specified in a previous NHANES analysis [1], testing for health effects of hyponatremia and/or underhydration, conditions that would be flagged by the hydration criteria.

Researchers agree that serum osmolality and its main component, serum sodium, are sensitive biomarkers of total body water deficit. With every 2% body weight loss via sweat during exercise, plasma osmolality increases about 5 mmol/kg [85]. Researchers further agree that serum osmolality and sodium have limited variability under conditions of daily life due to tight homeostatic regulation [55,86]. Researchers disagree, however, about relying on a biomarker that has limited variability under conditions of daily life to index hydration in people who are not hospitalized or not performing extreme exercise [86]. By itself, a normal serum osmolality or sodium can mask variability in metabolic and/or physiologic compensation. It does not discriminate individuals who have normal serum osmolality and/or normal serum sodium, thanks to urine concentration, from individuals who have normal values with no compensation required. Beyond the potential for unmeasured heterogeneity due to homeostatic compensation, use of serum osmolality or serum sodium to index hydration is complicated by the narrow 10 mmol/L normal range, variation in the set-point for homeostasis, and U-shaped relationship with health outcomes. A one-unit difference in serum osmolality or sodium may be clinically insignificant or only reflect measurement error. Cutoffs for defining the optimal normal range for these biomarkers may differ within and between individuals because the homeostatic set-point for serum osmolality or sodium can vary [61,87]. As hypo- and hypernatremia are both associated with adverse health outcomes, including death [10,88], use of continuous serum osmolality and serum sodium in epidemiologic analysis inappropriately masks U-shaped effects. Indeed, continuous serum osmolality is not linearly associated with mortality in NHANES data [89].

Researchers agree that spot urine osmolality is sensitive to acute and mild changes in fluid intake in the short-term [55,86], but disagree about using this biomarker to index ‘usual’ status in epidemiologic hydration studies. Despite disagreement, spot urine osmolality at a given time of day may be relatively stable among free-living individuals, as free-living individuals have stable within-person 24 h urine volume and osmolality, as well as intransigent fluid intake patterns [90]. Community-dwelling individuals who report lower fluid intake have elevated antidiuretic hormone (arginine vasopressin, AVP) and higher urine osmolality [57]. In non-acutely ill adults, following a sustained increase in total daily water intake (i.e., a change in the ‘usual’ level of water intake), spot urine osmolality decreases for all urine collections across the day, including the morning fasting collection [90,91]. The hydration classification used in this analysis was selected to match that defined for a previous NHANES analysis [1]. The hydration classification represents a compromise between only using serum osmolality or serum sodium and only using urine osmolality. It also represents an alignment of epidemiological research on free-living non-acutely ill populations with clinical differential diagnosis of fluid disorders in hospitalized patients [92].

### 4.2. Cross-Sectional Association between Hydration Classification and Chronic Health Conditions

The relationship between hydration classification and chronic health condition was described from two points of view, with hydration classification treated as either the dependent or independent variable. One or more chronic health condition was cross-sectionally associated with not meeting the hydration criteria and underhydration, more specifically. Conversely, not meeting hydration criteria and underhydration were respectively associated with chronic health conditions. Although a statistically significant association in both directions is anticipated when a statistically significant association is observed in one direction, the study provides weighted estimates of the proportion exposed and magnitudes of association for each direction of effect for U.S. adults aged 51–70 years. Population-representative estimates of each effect may be useful for future meta-analysis, causal inference do-calculus, program planning, or policy making.

Independent of covariates, obesity, high waist circumference, and insulin resistance each respectively increased the prevalence of not meeting the hydration criteria by 20%. Low HDL cholesterol increased the prevalence of not meeting the hydration criteria by 15%. The prevalence ratio for not meeting the hydration criteria was 15% higher for individuals with metabolic syndrome compared to individuals without metabolic syndrome. The results suggest need or opportunity for clinicians to systematically assess hydration for individuals with conditions of the metabolic syndrome, and chronic disease researchers to consider hydration as a potential confounder and/or effect modifier for chronic disease morbidity and mortality. The National Academy of Medicine report recognizes that illness increases water requirements [71]. Further research is needed to determine what level of water intake reduces the risk of underhydration for individuals with metabolic syndrome.

With hydration classification treated as the independent variable, the results suggest that not meeting the hydration criteria and underhydration, more specifically, have public health significance. Not meeting the hydration criteria and underhydration were associated with significantly increased prevalence of obesity, insulin resistance, diabetes, dyslipidemia, hypertension, and metabolic syndrome. Independent of age, sex, race/ethnicity, height, education, poverty level, physical activity, dietary solute load, total energy intake, carbohydrate intake, past or current cigarette smoking, prescription medication use, season, and length of fast, the proportion of people without any of the aforementioned conditions was 39% lower for individuals who did not meet hydration criteria compared to individuals who did meet the hydration criteria.

### 4.3. Potential for Endogenous Effects of Hydration and Chronic Disease

The results of this study were consistent with a potential negative feedback mechanism. Excepting the association between obesity and hydration classification, control for all other chronic health conditions explained away each observed association, signaling that the relationship between each health condition and hydration could conceivably be mediated by metabolic dysregulation due to other chronic conditions. Alternatively, each association might be confounded by one or more other health condition(s). If metabolic dysregulation mediates the relationship between each health condition and hydration, then metabolic dysregulation and the health conditions that are upstream to the metabolic dysregulation should not be controlled in multivariable analysis [93], but rather treated as a joint exposure. If, on the other hand, one or more health conditions confounds the association, explaining the relationship by a pathway that does not involve the index health condition, then stratified analyses would be needed to test for each association among those with no other condition. Longitudinal data with carefully selected study populations are needed to determine if each chronic health condition prospectively predicts underhydration.

The finding that obesity remained cross-sectionally associated with hydration classification after control for all other health conditions might suggest that this relationship preceded associations between underhydration and the other health conditions assessed in this analysis. Obesity might be a cause or consequence of underhydration. Large body size increases water intake requirements and likelihood of inadequate water intake. On the other hand, lower absolute and relative intake of drinking water have been observed to reduce energy expenditure, reduce fat oxidation, and induce insulin resistance, which contribute to positive fat balance [94]. Obesity reflects a sustained period of positive fat balance, due primarily to reduced oxidation of fat consumed [95,96]. The residual association that remained after control for other health conditions might also reflect measurement or misclassification error related to the method and cutoffs used to assess each health condition. Individuals with insulin resistance detectable by a HOMA-IR value of 2.5 may be more likely to already be obese than individuals with a milder HOMA-IR of 1.5. Longitudinal data and carefully defined health conditions would be required to determine the order of associations.

### 4.4. Potential Causal Mechanisms Linking Hydration with Metabolic Syndrome

Underhydration, that is, having a serum sodium above the normal range, low urine volume and/or concentrated urine, implies osmotic stress on cells. Osmotic stress on cells is a plausible causal factor for the metabolic syndrome, which is characterized by multiple simultaneous health conditions, as osmotic cell shrinkage acts as a metabolic switch that simultaneously alters multiple biochemical pathways, cellular processes, and physiological systems [29].

Longitudinal data suggest parallel relationships between various correlates of osmotic stress on cells and risk of the metabolic syndrome. *Behavioral* determinants of hyperosmotic stress on cells, such as salt intake, lower absolute water intake, and intake of hypertonic beverages such as sugar-sweetened beverages instead of drinking water [97,98,99,100,101]; *blood biomarker* measures of hyperosmotic stress on cells, such as serum hypernatremia, hypertonicity, or hyperosmolality [26,32,33,38,88,102]; intracellular measures of biochemical response to hyperosmotic cell shrinkage such as upregulation of the serum- and glucocorticoid-inducible kinase 1 (SGK1) [103,104]; and *physiologic* measures of response to hyperosmotic cell shrinkage, such as increases in plasma copeptin [18,19,105,106,107,108], are independently associated with incident metabolic dysregulation and/or chronic disease risk.

The parallel results suggest that osmotic stress on cells or some correlate(s) of osmotic stress on cells may be causally related to metabolic dysregulation and chronic disease risk. Any one or more measure might conceivably be the necessary and sufficient etiologic factor(s) for metabolic syndrome. Given that osmotic cell shrinkage simultaneously impacts multiple biochemical pathways, multiple cellular mechanisms may mediate effects of suboptimal fluid intake on pathophysiology and life span. Additional experimental effort is needed to unravel the molecular pathophysiology of the relationship between not meeting hydration criteria, underhydration, and chronic health conditions. The success of disease prevention and treatment efforts depends on identification of the necessary and sufficient causal factor(s).

### 4.5. Longitudinal Association between Hydration Classification and Mortality

The above paragraphs consider the potential for endogenous, interactive effects of hydration and health conditions on disease progression, as well as the potential for health conditions to simply confound effects of hydration on disease progression. If the underlying mechanism(s) involve(s) feedback of endogenous effects, then an individual’s stage in the feedback cycle at baseline would be an appropriate exposure for longitudinal mortality analysis. The effect to be estimated would be the joint effect of the hydration classification and chronic condition on mortality risk. If, on the other hand, baseline chronic health condition confounds the effect of hydration on disease progression, then the longitudinal effect to estimate would be the effect of hydration among individuals without chronic health conditions at baseline.

In the present analysis, the combination of one or more chronic health conditions in 2009–2012 and underhydration in 2009–2012 was associated with significantly greater all-cause and chronic disease mortality compared to the combination of one or more chronic conditions and euhydration in 2009–2012. The data are consistent with sub-optimal hydration exacerbating metabolic dysregulation and accelerating disease progression. It was not possible to estimate the effect of underhydration on mortality among those without a chronic health condition at baseline, as only five deaths occurred in this group. Zero chronic disease associated deaths were observed for people who did not have any of the chronic conditions and, also, met the hydration criteria in 2009–2012.

The results of the present NHANES analysis are consistent with the results of Mohan et al. [10] and inconsistent with those reported by Kant and Graubard [89]. Mohan et al. [10] report significantly greater mortality associated with hypernatremia vs. normonatremia in NHANES participants followed from 1999–2004 through December 31, 2006. They also report significantly greater mortality associated with hyponatremia vs. normonatremia. The U-shaped relationship between serum sodium and mortality may explain why Kant and Graubard [89] observed no linear association between continuous serum osmolality and all-cause mortality with up to 13 years of follow-up. The present sensitivity analyses confirmed a significant U-shaped relationship between serum osmolality and mortality in the 2009–2012 NHANES data (see Appendix). Null effects of individual hydration parameters on mortality may also reflect the fact that no single hydration measure isolates optimal hydration for non-acutely ill individuals. Normal serum osmolality may mask heterogeneity due to homeostatic compensation. Low urine osmolality may mask hyponatremia. The present sensitivity analyses confirmed significant variability in mortality associated with underhydration (low urine volume or urine osmolality above 500 mmol/kg) for individuals with a serum osmolality in the normal range, between 285–295 mmol/kg.

### 4.6. Confounding

Effects of hydration on the metabolic syndrome and chronic disease risk may be confounded by determinants of water requirements, determinants of water intake, and determinants of water handling in the body. Time invariant and time-varying environmental, socio-economic, behavioral, psychological, and biological factors may determine an individual’s water requirements and/or water intake at a given point in time. Environmental factors may include ambient temperature; altitude; the availability, accessibility, and quality or palatability of water; and place-based conditions, such as school or workplace policy (e.g., vending machine rules, recess or toilet breaks). Socio-economic factors may include cultural norms or ethnicity, education, and poverty. Behavioral factors include physical activity, solute intake, sleep, smoking, alcohol intake, and medication use. Psychological factors include stress and pain. Biological factors include age, sex, body size, race, metabolic adaptation (e.g., to habitual caffeine intake or heat stress), renal concentrating/diluting capacity, disease related factors (e.g., insult accumulation/wear and tear on organ systems, glycosuria), and genetic factors.

The present cross-sectional analysis was able to control for some environmental, socio-economic, behavioral, and genetic factors, but data were not available to also control for psychological factors. Stress and pain may confound the observed associations because stress and pain can trigger vasopressin and cortisol release [109,110], which stimulate urine concentration. At the same time, stress and pain induce metabolic shifts that favor the flight or fight response, a profile of metabolic dysregulation that is characteristic of the metabolic syndrome. In addition to better control for environmental and psychological factors, future prospective studies of hydration effects on chronic disease risk should address time-varying factors.

Variables which might confound the observed cross-sectional associations between hydration and chronic health conditions may also confound the observed longitudinal relationships between hydration and mortality. In the present study, longitudinal models predicting death within 3–6 years controlled for the same covariates addressed in the cross-sectional analysis. On the basis of the hypothesized causal mechanism illustrated in Figure 1, the analysis did not control for all possible confounding variables.

The present study observed that spot urine volume below 50 mL was associated with significantly increased mortality risk. This finding, considered with the association between serum hypernatremia and increased mortality reported by Mohan et al. [10], suggests that the observed association between underhydration and increased mortality is not solely attributable to urine osmolality of 500 mmol/kg or more due to uncontrolled diabetes-related glycosuria. Urine glucose data were not available to rule out a confounding by glycosuria. Future work to confirm and characterize an independent effect of underhydration on mortality should consider potential confounding by glycosuria.

Acknowledging that observational data might be informative regarding causal effects, even when confounding variables are unmeasured [93], the present analysis reported weighted probabilities of having health conditions and estimated number of deaths, in addition to relative hazard ratios, in case readers wish to apply front door or do-calculus techniques to estimate the probability of death caused by underhydration. Front door adjustment may not be applicable to the present estimation problem, however, if the unmeasured confounders (e.g., environmental factors, pain, psychological stress) have a strong effect on the mediating metabolic dysregulation. Independence of the intermediate from the unobserved confounder is a prerequisite for using front door adjustment [93]. The lack of data regarding metabolic dysregulation from an intermediate time point limits the approach. Information or assumptions about a weak, negligible effect of environmental and psychological factors on metabolic dysregulation, which remains consistent over time, in the population of interest might enable front door adjustment.

### 4.7. Analysis Limitations

The present analysis was limited by the non-randomized, observational design, lack of serial measures of hydration biomarkers, chronic health conditions, relatively short 3 to 6 year follow-up period and small number of deaths. Although chronic health condition was determined from laboratory tests and multivariable models were used to control for confounding, the results are vulnerable to measurement or misclassification error and residual confounding. Interpretation of the results is limited to descriptive purposes and hypothesis generation about underlying causal mechanisms.

The analysis defined hydration in terms of biomarkers, which are sensitive to hypertonic dehydration but may be relatively insensitive to isotonic dehydration and/or hypohydration for persons with deficits in urine concentrating ability. The hydration criteria may have limited interpretation for individuals with advanced chronic disease. Serum sodium and urine osmolality are influenced by chronic health conditions. Hyperglycemia can distort serum sodium downwards. Urine glucose may distort urine osmolality upwards. Medications prescribed for chronic disease, such as diuretics, may disassociate serum sodium and urine osmolality from dietary intake. Renal urine concentration and/or urine dilution capacity may influence urine osmolality. The present sensitivity analyses confirmed that older age significantly modifies the effect of underhydration on chronic disease mortality.

### 4.8. Future Directions

In addition to the question of whether or not underhydration truly causes an increased risk of chronic disease and mortality, the present results raise questions about what type and amount of water intake could reduce said risk. In the preceding NHANES analysis, the prevalence of not meeting hydration criteria was significantly higher for non-acutely ill men and women aged 51–70 years with daily total water intake (TWI) below 45 mL/kg or plain drinking water intake (PWI) below 20 mL/kg [1]. The discussion of that paper calls for careful sensitivity and specificity analyses to identify the optimal ml/kg TWI and PWI for each population sub-group and condition. Group- and condition-specific randomized trials are needed to test effects of different levels of water intake, defined relative to optimal ml/kg TWI or PWI cutoffs, on chronic disease incidence and progression.

## 5. Conclusions

The present study extends inference regarding the reported high prevalence of not meeting hydration criteria among U.S. adults aged 51–70 years [1]. The study provides nationally representative estimates of association between not meeting the hydration criteria and the prevalence of chronic health conditions. Despite the sample size limitations, the analysis reveals a statistically significant decrease in survival, within 3 to 6 years, for adults aged 51–70 years with a pre-existing chronic health condition who did not meet the hydration criteria, and were underhydrated, more specifically. The results draw attention to the public health impact of not meeting hydration criteria.

## Figures and Tables

**Table 1:** Characteristics of the study population in 2009–2012.

			Euhydration (*n* = 319)	Did Not Meet Hydration Criteria (*n* = 881)	
		*n*	Weighted % (95% CI)	Weighted % (95% CI)	*p*-Value
All	-	1200	28.6 (24.6–33.0)	71.4 (67.0–75.4)	-
Age, years	51–60	654	29.3 (24.5–34.7)	70.7 (65.3–75.5)	Reference
	61–70	546	27.5 (21.7–34.3)	72.5 (65.7–78.3)	0.629
Sex	Female	609	31.3 (25.7–37.4)	68.7 (62.6–74.3)	0.112
	Male	591	25.5 (20.9–30.9)	74.5 (69.1–79.1)	Reference
Height, cm	<167	609	31.2 (25.8–37.2)	68.8 (62.8–74.2)	Reference
	≥167	591	26.4 (21.4–32.2)	73.6 (67.8–78.6)	0.179
Race/ethnicity	Black	288	21.2 (16.6–26.6)	78.8 (73.4–83.4)	0.011
	Hispanic	312	22.2 (17.8–27.3)	77.8 (72.7–82.2)	0.011
	Other	99	45.1 (31.5–59.4)	54.9 (40.6–68.5)	Reference
	White	501	29.3 (24.2–35.0)	70.7 (65.0–75.8)	0.052
Education	<High school	321	24.0 (17.3–32.3)	76.0 (67.7–82.7)	0.098
	High school	584	27.9 (22.8–33.5)	72.1 (66.5–77.2)	0.327
	College	295	31.9 (25.3–39.4)	68.1 (60.6–74.7)	Reference
Poverty, % of FPL	Unknown	112	33.1 (20.3–49.0)	66.9 (51.0–79.7)	0.724
	<100	195	31.3 (22.9–41.1)	68.7 (58.9–77.1)	0.819
	100–199	271	28.8 (22.7–35.7)	71.2 (64.3–77.3)	0.788
	200–399	265	23.6 (17.7–30.6)	76.4 (69.4–82.3)	0.216
	400+	469	30.5 (24.1–37.7)	69.5 (62.3–75.9)	Reference
Physical activity, MET min/w	<1000	637	30.4 (25.7–35.6)	69.6 (64.4–74.3)	Reference
	1000–4999	368	27.3 (21.0–34.7)	72.7 (65.3–79.0)	0.399
	≥5000	195	26.1 (19.7–33.6)	73.9 (66.4–80.3)	0.258
Cigarette smoking	Never	591	30.1 (24.9–35.8)	69.9 (64.2–75.1)	Reference
	Past	402	20.5 (15.5–26.5)	79.5 (73.5–84.5)	0.006
	Current	207	42.8 (33.3–52.9)	57.2 (47.1–66.7)	0.020
Alcohol, drinks/day	0	885	27.7 (23.1–32.9)	72.3 (67.1–76.9)	Reference
	1–2	183	33.7 (28.1–39.8)	66.3 (60.2–71.9)	0.101
	Over 2	132	26.5 (16.9–38.9)	73.5 (61.1–83.1)	0.818
Dietary solute load, mOsm/day	<1000	1049	30.1 (26.0–34.5)	69.9 (65.5–74.0)	Reference
	≥1000	151	19.1 (11.0–31.0)	80.9 (69.0–89.0)	0.019
Total energy, kcal/day	<1860	599	35.5 (30.3–41.1)	64.5 (58.9–69.7)	Reference
	≥1860	601	22.9 (17.6–29.2)	77.1 (70.8–82.4)	0.002
Total carbohydrate, % of energy	<50	623	23.3 (18.9–28.4)	76.7 (71.6–81.1)	Reference
	≥50	577	35.4 (29.0–42.4)	64.6 (57.6–71.0)	0.005
Medication use	No	332	26.3 (19.5–34.6)	73.7 (65.4–80.5)	Reference
	Yes	868	29.4 (24.6–34.6)	70.6 (65.4–75.4)	0.507
Season	Spring–summer	656	25.8 (20.6–31.6)	74.2 (68.4–79.4)	Reference
	Fall–winter	544	33.3 (26.6–40.7)	66.7 (59.3–73.4)	0.105
Length of fast, h	8–11	319	26.5 (21.8–31.8)	73.5 (68.2–78.2)	Reference
	12–22	881	30.6 (25.5–36.2)	69.4 (63.8–74.5)	0.175

Weighted estimates are representative of fasting U.S. adults aged 51–70 years in 2009–2012. Euhydration: serum sodium 135–144 mmol/L, urine osmolality below 500 mmol/kg, and urine volume 50 mL or higher. Did not meet hydration criteria: Did not meet one or more criteria for euhydration. FPL: Federal Poverty Level; MET: metabolic equivalent. Groups were compared in unadjusted Poisson models with robust error variance.

**Table:** (**A**)

Chronic Condition	Had Chronic Health Condition	NHANES Participants Who Did Not Meet Criteria	U.S. Adults Aged 51–70 Years Who Did Not Meet Criteria	Prevalence Ratio for Not Meeting the Hydration Criteria					
				Unadjusted		Multivariable Adjusted			
				Model 1		Model 2		Model 3	
		*n*	Weighted % (95% CI)	Weighted PR (95% CI)	*p*	Weighted PR (95% CI)	*p*	Weighted PR (95% CI)	*p*
Obesity	No	488	63.8 (57.1–70.1)	1.0		1.0		1.0	
	Yes	393	83.0 (77.5–87.3)	1.30 (1.14–1.48)	0.000	1.23 (1.10–1.38)	0.001	1.18 (1.05–1.34)	0.009
High waist circumference	No	294	60.2 (52.7–67.2)	1.0		1.0		1.0	
	Yes	587	76.8 (72.1–80.9)	1.28 (1.13–1.44)	0.000	1.23 (1.08–1.41)	0.003	1.09 (0.95–1.25)	0.200
Insulin resistance	No	716	68.9 (64.3–73.1)	1.0		1.0		1.0	
	Yes	165	86.9 (78.0–92.5)	1.26 (1.14–1.40)	0.000	1.20 (1.09–1.32)	0.000	1.08 (0.97–1.20)	0.160
Diabetes	No	641	69.5 (65.1–73.6)	1.0		1.0		1.0	
	Yes	240	78.9 (70.4–85.4)	1.14 (1.03–1.25)	0.012	1.09 (0.99–1.21)	0.092	1.01 (0.90–1.12)	0.905
Elevated glucose	No	742	70.1 (66.0–74.0)	1.0		1.0		1.0	
	Yes	139	80.8 (69.0–88.8)	1.15 (1.03–1.29)	0.015	1.07 (0.93–1.23)	0.311	0.99 (0.86–1.15)	0.944
Elevated HbA1c	No	745	70.3 (65.9–74.3)	1.0		1.0		1.0	
	Yes	136	79.5 (67.6–87.7)	1.13 (0.99–1.29)	0.062	1.07 (0.93–1.24)	0.340	0.97 (0.83–1.13)	0.655
Elevated TG or low HDL	No	541	68.9 (63.2–74.0)	1.0		1.0		1.0	
	Yes	340	75.3 (70.0–79.9)	1.09 (1.00–1.20)	0.058	1.10 (1.01–1.20)	0.038	1.03 (0.93–1.14)	0.526
Elevated TG	No	643	70.5 (65.4–75.1)	1.0		1.0		1.0	
	Yes	238	73.7 (67.1–79.5)	1.05 (0.95–1.15)	0.353	1.06 (0.97–1.16)	0.224	1.00 (0.91–1.09)	0.932
Low HDL	No	654	68.8 (63.5–73.7)	1.0		1.0		1.0	
	Yes	227	79.2 (71.7–85.1)	1.15 (1.02–1.29)	0.021	1.15 (1.01–1.31)	0.033	1.09 (0.95–1.26)	0.204
Hypertension	No	199	63.7 (54.7–71.8)	1.0		1.0		1.0	
	Yes	682	74.2 (69.6–78.4)	1.17 (1.01–1.34)	0.031	1.13 (0.98–1.29)	0.089	1.07 (0.94–1.22)	0.271
Metabolic syndrome	No	593	67.7 (62.5–72.6)	1.0		1.0		1.0	
	Yes	288	80.4 (73.4–85.9)	1.19 (1.07–1.32)	0.002	1.15 (1.05–1.27)	0.006	-	-
None of the above	No	806	73.4 (68.9–77.5)	1.0		1.0		-	
	Yes	75	55.8 (45.4–65.6)	0.76 (0.64–0.90)	0.003	0.80 (0.66–0.96)	0.021	-	-

The table reports data from the 2009–2012 National Health and Nutrition Examination Survey (NHANES). With survey weights applied, estimates are representative of fasting U.S. adults aged 51–70 years in 2009–2012. Participants were classified as meeting the hydration criteria if they had a serum sodium greater than or equal to 135 and less than 145 mmol/L, a urine volume greater than or equal to 50 mL, and a urine osmolality below <500 mmol/kg. The prevalence ratio for not meeting the hydration criteria was estimated using Poisson models with robust error variances and survey weights applied. Model 1 did not adjust for covariates. Model 2 controlled for age, sex, height, race/ethnicity, education, poverty level, physical activity, total energy intake, total carbohydrate intake, alcoholic drinks consumed, dietary solute load, past or current cigarette smoking, prescription medication use, season of mobile examination center (MEC) measurement and length of fast. Model 3 added control for all other chronic health conditions in the table.

**Table:** (**B**)

Chronic Condition	Had Chronic Health Condition	NHANES Participants Who Were Underhydrated	U.S. Adults Aged 51–70 Years Who Were Underhydrated	Prevalence Ratio for Underhydration vs. Euhydration					
				Unadjusted Model 1		Multivariable Adjusted			
						Model 2		Model 3	
		*n*	Weighted % (95% CI)	Weighted PR (95% CI)	*p*	Weighted PR (95% CI)	*p*	Weighted PR (95% CI)	*p*
Obesity	No	713	62.6 (56.1–68.7)	1.0		1.0		1.0	
	Yes	487	79.9 (74.3–84.5)	1.30 (1.14–1.49)	0.000	1.24 (1.10–1.39)	0.001	1.19 (1.05–1.35)	0.007
High waist circumference	No	439	58.8 (51.6–65.5)	1.0		1.0		1.0	
	Yes	761	74.6 (70.2–78.5)	1.28 (1.14–1.44)	0.000	1.24 (1.08–1.42)	0.003	1.09 (0.96–1.25)	0.181
Insulin resistance	No	1003	67.5 (63.0–71.7)	1.0		1.0		1.0	
	Yes	197	81.4 (73.7–87.3)	1.26 (1.14–1.39)	0.000	1.19 (1.09–1.31)	0.000	1.07 (0.96–1.19)	0.196
Diabetes	No	892	68.1 (63.8–72.2)	1.0		1.0		1.0	
	Yes	308	74.6 (67.5–80.5)	1.13 (1.02–1.24)	0.016	1.09 (0.98–1.20)	0.118	1.01 (0.90–1.13)	0.906
Elevated glucose	No	1030	68.7 (64.6–72.6)	1.0		1.0		1.0	
	Yes	170	74.7 (64.0–83.1)	1.14 (1.02–1.28)	0.025	1.06 (0.92–1.22)	0.398	0.99 (0.85–1.16)	0.917
Elevated HbA1c	No	1028	69.0 (64.7–73.0)	1.0		1.0		1.0	
	Yes	172	72.6 (61.0–81.7)	1.11 (0.97–1.27)	0.110	1.06 (0.91–1.23)	0.463	0.96 (0.81–1.13)	0.605
Elevated TG or low HDL	No	744	67.6 (62.1–72.7)	1.0		1.0		1.0	
	Yes	456	72.3 (67.7–76.5)	1.09 (0.99–1.19)	0.073	1.10 (1.01–1.20)	0.039	1.04 (0.93–1.15)	0.486
Elevated TG	No	883	69.3 (64.3–73.9)	1.0		1.0		1.0	
	Yes	317	69.8 (64.5–74.6)	1.04 (0.94–1.14)	0.460	1.05 (0.97–1.15)	0.237	1.00 (0.91–1.09)	0.970
Low HDL	No	898	66.8 (61.8–71.5)	1.0		1.0		1.0	
	Yes	302	77.5 (70.8–83.0)	1.16 (1.03–1.30)	0.018	1.15 (1.01–1.32)	0.034	1.10 (0.95–1.27)	0.192
Hypertension	No	289	63.4 (54.4–71.6)	1.0		1.0		1.0	
	Yes	911	71.7 (67.4–75.6)	1.16 (1.00–1.33)	0.044	1.12 (0.97–1.29)	0.125	1.06 (0.93–1.21)	0.338
Metabolic syndrome	No	836	66.7 (61.4–71.5)	1.0		1.0		1.0	
	Yes	364	76.3 (69.4–82.1)	1.18 (1.06–1.31)	0.003	1.15 (1.04–1.28)	0.008	-	-
None of the above	No	1084	71.2 (67.3–74.8)	1.0		1.0		-	
	Yes	116	55.4 (45.1–65.3)	0.76 (0.64–0.91)	0.000	0.81 (0.67–0.97)	0.027	-	-

The table reports data from the 2009–2012 National Health and Nutrition Examination Survey (NHANES). With survey weights applied, estimates are representative of fasting U.S. adults aged 51–70 years in 2009–2012. Participants were classified as underhydrated if they had a serum sodium greater than or equal to 145 mmol/L, a urine volume less than 50 mL, or a urine osmolality greater than or equal to 500 mmol/kg. The prevalence ratio for underhydration was estimated using Poisson models with robust error variances and survey weights applied. Model 1 did not adjust for covariates. Model 2 controlled for age, sex, height, race/ethnicity, education, poverty level, physical activity, total energy intake, total carbohydrate intake, alcoholic drinks consumed, dietary solute load, past or current cigarette smoking, prescription medication use, season of MEC measurement, and length of fast. Model 3 added control for all other chronic health conditions in the table.

**Table:** (**A**)

Chronic Condition	Met Hydration Criteria	NHANES Participants with Condition	U.S. Adults Aged 51–70 Years with Condition	Prevalence Ratio for Having the Chronic Health Condition					
				Unadjusted		Multivariable Adjusted			
				Model 1		Model 2		Model 3	
		*n*	Weighted % (95% CI)	Weighted PR (95% CI)	*p*	Weighted PR (95% CI)	*p*	Weighted PR (95% CI)	*p*
Obesity	No	393	45.9 (39.8–52.2)	1.95 (1.38–2.77)	0.000	1.72 (1.26–2.37)	0.001	1.50 (1.11–2.03)	0.010
	Yes	94	23.5 (17.3–31.1)	1.0		1.0		1.0	
High waist circumference	No	587	72.6 (67.4–77.1)	1.33 (1.16–1.52)	0.000	1.25 (1.08–1.44)	0.004	1.09 (0.95–1.25)	0.200
	Yes	174	54.7 (46.9–62.3)	1.0		1.0		1.0	
Insulin resistance	No	165	16.9 (12.8–22.0)	2.66 (1.47–4.83)	0.002	2.29 (1.33–3.97)	0.004	1.55 (1.02–2.36)	0.042
	Yes	32	6.4 (3.5–11.2)	1.0		1.0		1.0	
Diabetes	No	240	22.1 (17.9–27.0)	1.50 (1.04–2.15)	0.030	1.31 (0.91–1.88)	0.146	1.07 (0.73–1.55)	0.731
	Yes	68	14.8 (10.5–20.3)	1.0		1.0		1.0	
Elevated glucose	No	139	13.2 (9.9–17.4)	1.68 (0.99–2.87)	0.055	1.32 (0.74–2.36)	0.328	1.06 (0.56–1.98)	0.859
	Yes	31	7.9 (4.7–12.9)	1.0		1.0		1.0	
Elevated HgA1c	No	136	13.0 (9.9–16.9)	1.55 (0.90–2.68)	0.113	1.34 (0.72–2.50)	0.342	0.96 (0.51–1.79)	0.886
	Yes	36	8.4 (5.4–12.8)	1.0		1.0		1.0	
Elevated TG or low HDL	No	340	40.7 (35.9–45.7)	1.22 (0.99–1.51)	0.065	1.25 (1.01–1.55)	0.040	1.06 (0.84–1.34)	0.590
	Yes	116	33.3 (26.9–40.5)	1.0		1.0		1.0	
Elevated TG	No	238	28.9 (24.3–34.0)	1.12 (0.87–1.50)	0.365	1.16 (0.91–1.48)	0.214	0.98 (0.77–1.25)	0.856
	Yes	79	25.7 (20.6–31.5)	1.0		1.0		1.0	
Low HDL	No	227	27.2 (23.2–31.5)	1.52 (1.03–2.26)	0.036	1.53 (1.00–2.32)	0.049	1.27 (0.82–1.98)	0.279
	Yes	75	17.9 (12.3–25.2)	1.0		1.0		1.0	
Hypertension	No	682	75.9 (70.9–80.2)	1.16 (1.02–1.31)	0.026	1.12 (0.99–1.28)	0.080	1.07 (0.96–1.21)	0.224
	Yes	229	65.7 (57.7–72.8)	1.0		1.0		1.0	
Metabolic syndrome	No	288	32.4 (27.9–37.3)	1.64 (1.16–2.31)	0.006	1.55 (1.10–2.17)	0.013	1.17 (0.92–1.50)	0.199
	Yes	76	19.8 (14.1–27.0)	1.0		1.0		1.0	
None of the above	No	75	8.9 (6.4–12.3)	0.52 (0.37–0.73)	0.000	0.61 (0.38–0.96)	0.035	-	-
	Yes	41	17.6 (12.5–24.3)	1.0		1.0		-	-

The table reports data from the 2009–2012 National Health and Nutrition Examination Survey (NHANES). With survey weights applied, estimates are representative of fasting U.S. adults aged 51–70 years in 2009–2012. Participants were classified as meeting the hydration criteria if they had a serum sodium greater than or equal to 135 and less than 145 mmol/L, a urine volume greater than or equal to 50 mL, and a urine osmolality below <500 mmol/kg. The prevalence ratio for each chronic health condition was estimated using Poisson models with robust error variances and survey weights applied. Model 1 did not adjust for covariates. Model 2 controlled for age, sex, height, race/ethnicity, education, poverty level, physical activity, total energy intake, total carbohydrate intake, alcoholic drinks consumed, dietary solute load, past or current cigarette smoking, prescription medication use, season of MEC measurement, and length of fast. Model 3 added control for all other chronic conditions in the table.

**Table:** (**B**)

Chronic Condition	Under-Hydrated	NHANES Participants with Condition	US Adults Aged 51–70 Years with Condition	Prevalence Ratio for Having the Chronic Health Condition					
				Unadjusted		Multivariable Adjusted			
				Model 1		Model 2		Model 3	
		*n*	Weighted % (95% CI)	Weighted PR (95% CI)	*p*	Weighted PR (95% CI)	*p*	Weighted PR (95% CI)	*p*
Obesity	No	94	26.0 (19.2–34.1)	1.93 (1.36–2.74)	0.001	1.71 (1.25–2.35)	0.002	1.50 (1.11–2.04)	0.010
	Yes	384	45.5 (39.4–51.7)	1.0		1.0		1.0	
High waist circumference	No	174	54.7 (46.9–62.3)	1.32 (1.15–1.52)	0.000	1.24 (1.07–1.44)	0.005	1.09 (0.95–1.25)	0.193
	Yes	573	72.4 (67.3–77.1)	1.0		1.0		1.0	
Insulin resistance	No	32	6.4 (3.5–11.2)	2.56 (1.45–4.53)	0.002	2.21 (1.31–3.75)	0.004	1.54 (1.02–2.32)	0.041
	Yes	158	16.3 (13.0–20.3)	1.0		1.0		1.0	
Diabetes	No	68	14.8 (10.5–20.3)	1.46 (1.03–2.06)	0.035	1.27 (0.88–1.82)	0.187	1.05 (0.72–1.54)	0.776
	Yes	231	21.5 (18.1–25.4)	1.0		1.0		1.0	
Elevated glucose	No	31	7.9 (4.7–12.9)	1.60 (0.96–2.66)	0.068	1.26 (0.71–2.22)	0.416	1.03 (0.55–1.93)	0.915
	Yes	133	12.6 (10.0–15.7)	1.0		1.0		1.0	
Elevated HgA1c	No	36	8.4 (5.4–12.8)	1.46 (0.85–2.49)	0.163	1.25 (0.67–2.33)	0.465	0.93 (0.49–1.75)	0.813
	Yes	129	12.2 (9.9–15.0)	1.0		1.0		1.0	
Elevated TG or low HDL	No	116	33.3 (26.9–40.5)	1.21 (0.98–1.49)	0.078	1.24 (1.00–1.53)	0.049	1.06 (0.84–1.34)	0.612
	Yes	329	40.2 (35.7–44.9)	1.0		1.0		1.0	
Elevated TG	No	79	25.7 (20.6–31.5)	1.09 (0.85–1.40)	0.466	1.13 (0.90–1.43)	0.278	0.97 (0.76–1.23)	0.771
	Yes	229	28.1 (24.0–32.6)	1.0		1.0		1.0	
Low HDL	No	75	17.9 (12.3–25.2)	1.53 (1.04–2.26)	0.033	1.53 (1.01–2.33)	0.046	1.28 (0.83–1.99)	0.258
	Yes	221	27.4 (23.6–31.5)	1.0		1.0		1.0	
Hypertension	No	229	65.7 (57.7–72.8)	1.15 (1.01–1.30)	0.037	1.11 (0.98–1.27)	0.109	1.07 (0.95–1.20)	0.261
	Yes	663	75.3 (69.9–80.0)	1.0		1.0		1.0	
Metabolic syndrome	No	76	19.8 (14.1–27.0)	1.60 (1.14–2.26)	0.008	1.51 (1.07–2.13)	0.021	1.17 (0.91–1.50)	0.209
	Yes	277	31.7 (27.1–36.6)	1.0		1.0		1.0	
None of the above	No	41	9.1 (6.5–12.6)	0.52 (0.37–0.73)	0.000	0.61 (0.38–0.96)	0.034	-	-
	Yes	74	17.6 (12.5–24.3)	1.0		1.0		-	-

The table reports data from the 2009–2012 National Health and Nutrition Examination Survey (NHANES). With survey weights applied, estimates are representative of fasting U.S. adults aged 51–70 years in 2009–2012. Participants were classified as underhydrated if they had a serum sodium greater than or equal to 145 mmol/L, a urine volume less than 50 mL, or a urine osmolality greater than or equal to 500 mmol/kg. The prevalence ratio for each chronic health condition was estimated using Poisson models with robust error variances and survey weights applied. Model 1 did not adjust for covariates. Model 2 controlled for age, sex, height, race/ethnicity, education, poverty level, physical activity, total energy intake, total carbohydrate intake, alcoholic drinks consumed, dietary solute load, past or current cigarette smoking, prescription medication use, season of MEC measurement, and length of fast. Model 3 added control for all other chronic conditions in the table.

**Table 4:** Number of deaths in 3 to 6 years to U.S. adults aged 51–70 years by hydration classification and chronic health condition.

Hydration Classification in 2009–2012	Chronic Health Condition in 2009–2012	Number of Deaths				Incidence Rate for U.S. Adults	
		NHANES Participants		U.S. Adults		Deaths per 1000 Person-Years	
		Number		Weighted estimate		Weighted estimate	
		All-Cause	Chronic Disease	All-Cause	Chronic Disease	All-Cause	Chronic Disease
All	All	52	33	1,724,051	1,084,144		
Euhydrated	All	11	5	255,230	92,733	3.1 (1.6–6.9)	1.1 (0.4–3.9)
Did not meet criteria		41	28	1,468,821	991,411	7.3 (5.0–11.1)	4.9 (3.2–8.2)
Hyponatremic		2	0	32,259	-	6.6 (0.5–34.6)	-
Underhydrated		39	28	1,436,562	991,411	7.3 (5.0–11.2)	5.1 (3.2–8.4)
Euhydrated	One or more	10	5	198,365	92,733	2.9 (1.5–6.3)	1.4 (0.5–4.7)
Did not meet criteria		37	25	1,328,598	863,305	7.3 (4.9–11.3)	4.7 (2.9–8.1)
Hyponatremic		2	0	32,259	-	6.8 (0.6–349.4)	-
Underhydrated		35	25	1,296,338	863,305	7.3 (4.9–11.4)	4.9 (3.0–8.3)
Euhydrated	None	1	0	56,865	-	-	-
Did not meet criteria		4	3	140,223	128,107	7.9 (2.0–52.7)	7.2 (1.6–64.5)
Hyponatremic		0	0	-	-	-	-
Underhydrated		4	3	140,223	128,107	7.9 (2.0–52.9)	7.2 (1.6–64.8)

National Health and Nutrition Examination Survey (NHANES) participants were followed through December 31, 2015 for all-cause and chronic disease-associated mortality. With survey weights applied, estimates are representative of fasting U.S. adults aged 51–70 years in 2009–2012. Euhydrated: participants had a serum sodium of 135–144 mmol/L, a urine volume greater than or equal to 50 mL, and a urine osmolality below 500 mmol/kg. Did not meet hydration criteria: participants who did not meet one or more criteria for euhydration were classified as not meeting hydration criteria. Hyponatremic: participants had a serum sodium below 135 mmol/L. Underhydrated: participants had a serum sodium of 145 mmol/L or higher, a urine volume below 50 mL, or a urine osmolality of 500 mmol/kg or higher. One or more: participants were classified as having one or more chronic health conditions if they had obesity, high waist circumference, insulin resistance, diabetes, elevated glucose, elevated HgA1c, elevated triglycerides, low HDL, hypertension, or metabolic syndrome in 2009–2012. None: participants were classified as having none of the chronic health conditions if they did not have any of the listed conditions in 2009–2012. The number of deaths and death rates with jackknife confidence intervals were estimated with NHANES survey weights applied.

## Appendix A

**Table A1:** Sensitivity analysis checking for a U-shaped relationship between serum osmolality and all-cause and chronic disease mortality in NHANES 2009–2012 participants aged 51–80 years.

Age	Number of NHANES Participants		Control for Covariates	Main Effect		Squared Term	
Years	Total	Deaths		Weighted HR (SE)	*p*-Value	Weighted HR (SE)	*p*-Value
All-cause mortality							
51–70	1200	52	Unadjusted	0.02 (0.03)	0.015	1.01 (0.003)	0.016
			Adjusted	0.02 (0.03)	0.020	1.01 (0.003)	0.020
51–80	1621	109	Unadjusted	0.01 (0.008)	0.000	1.01 (0.001)	0.000
			Adjusted	0.07 (0.05)	0.003	1.00 (0.001)	0.003
Chronic disease mortality							
51–70	1200	33	Unadjusted	0.03 (0.07)	0.170	1.01 (0.004)	0.163
			Adjusted	0.03 (0.11)	0.303	1.01 (0.01)	0.291
51–80	1621	63	Unadjusted	0.01 (0.01)	0.000	1.01 (0.002)	0.000
			Adjusted	0.04 (0.03)	0.000	1.01 (0.001)	0.000

National Health and Nutrition Examination Survey (NHANES). With survey weights applied, the estimated hazard ratios (HR) are representative of fasting U.S. adults aged 51–70 years in 2009–2012. Weighted Poisson models testing for a U-shaped effect of serum osmolality and mortality included a main effect term for serum osmolality and an interaction term (serum osmolality squared). Serum osmolality was calculated as 1.86 × (Na+ + K+) + 1.15 × glucose + urea + 14 [110]. The multivariable adjusted weighted models controlled for age in years, sex, height, race/ethnicity, poverty level, education level, physical activity level, total energy intake, dietary solute load, percent of total dietary energy from carbohydrate, number of alcoholic drinks, smoking, medication use, season, and hours of fast before measurement.

**Table A2:** Sensitivity analysis checking what range in the serum osmolality distribution contributed to the U-shaped relationship with chronic disease mortality for NHANES 2009–2012 participants aged 51–80 years.

Serum Osmolality	NHANES Participants	Chronic Disease	U.S. Adults Aged 51–70 Years	Hazard Ratio			
		Deaths					
mmol/kg	*n*	*n*	Weighted % (95% CI)	Unadjusted Weighted HR (95% CI)	*p*	Adjusted Weighted HR (95% CI)	*p*
<285	57	2	4.9 (3.6–6.7)	0.99 (0.23–4.31)	0.985	0.96 (0.19–4.94)	0.961
285–294	944	30	60.2 (57.3–63.1)	1.0		1.0	
> = 295	557	31	34.9 (31.4–38.6)	1.48 (0.83–2.63)	0.177	1.20 (0.71–2.05)	0.488
<280	12	1	1.0 (0.5–1.7)	2.01 (0.27–14.95)	0.483	1.74 (0.33–9.10)	0.501
280–299	1460	52	94.2 (92.7–95.4)	1.0		1.0	
> = 300	86	10	4.9 (3.8–6.4)	2.53 (1.21–5.29)	0.015	2.00 (1.01–3.96)	0.048

National Health and Nutrition Examination Survey (NHANES). With survey weights applied, estimates are representative of fasting U.S. adults aged 51–80 years in 2009–2012. Poisson models estimated the hazard ratio. The multivariable adjusted models controlled for age in years, sex, height, race/ethnicity, poverty level, education level, physical activity level, total energy intake, dietary solute load, percent of total dietary energy from carbohydrate, number of alcoholic drinks, smoking, medication use, season, and hours of fast before measurement.
